# Protecting public health in Europe: building trust in a challenging world

**DOI:** 10.2807/1560-7917.ES.2024.29.29.2400461

**Published:** 2024-07-18

**Authors:** Pamela Rendi-Wagner

**Affiliations:** 1 European Centre for Disease Prevention and Control (ECDC), Stockholm, Sweden

**Keywords:** public health, Europe, health threats, crisis

On 17 June 2024, I was pleased to take up my post as the fourth Director of the European Centre for Disease Prevention and Control (ECDC). Public health has long been an issue that is close to my heart. Already as a young doctor working in different parts of the world, I realised that while classical medicine is vitally important, the impact of taking action in public health is even broader. At the level of the European Union (EU), public health means working to protect and improve the lives of 500 million people.

We are currently faced with an increasingly challenging world that also has major implications on public health: a war in Europe, the accelerating effects of climate change, increasing social inequalities. War, flooding and the effects of climate change are all catalysts for infectious diseases. And when people believe misinformation over science, medical advances we take for granted can be swiftly reversed.

The COVID-19 pandemic was a stark illustration of the stakes. Countries’ preparedness plans were put to a test, a wide range of non-pharmaceutical measures were implemented, and the need for strong health systems and a resilient well-trained workforce became evident. Public funding and new technologies managed to rapidly develop safe and effective vaccines that were largely well received by citizens. However, parts of the population remained sceptical towards the various measures taken, including vaccination. Even before the pandemic, vaccine uptake was a challenge for politicians and scientists alike, and in several EU countries there were pockets of the population with low coverage for important childhood and adult vaccines. Understanding the reasons for this by actively engaging and communicating with communities and the general public, and continuously monitoring vaccine effectiveness and safety will be crucial to building trust in vaccines and working towards increasing vaccination coverage.

Reinforcing and restoring trust in science is one of the great challenges of our time, and it is particularly urgent in our field. When the next public health crisis comes, what if we can no longer distinguish between real speeches made by world leaders and those generated using artificial intelligence, for example? We need to tackle these issues now, using all the innovation and resources we can muster, before the situation becomes irretrievable. Above all, people want to feel secure in their lives – and ECDC’s mission is to ensure that happens in the field of public health and infectious diseases.

So how do we do that? During the pandemic, ECDC helped public health authorities across the EU make rapid decisions by providing accurate epidemiological data and timely risk assessments. But just as health systems and societies at large were placed under enormous pressure, so was ECDC. The difference between preparing for a pandemic and dealing with one in real life highlighted gaps, and as a result, in December 2022, the European Commission launched the European Health Union [1] with the aim of supporting EU countries to better anticipate, prepare, and respond to future health threats of all kinds: emergencies, outbreaks, new pathogens, antimicrobial resistance, and vaccine-preventable diseases. Two major building blocks of this were the strengthening of ECDC’s mandate [2] and the introduction of a new regulation on serious cross-border threats to health [3]. Taken together, they form the strategic outline for the agency’s core work in the coming months and years.

Implementation of the new mandate is well underway. ECDC will support countries in their preparedness activities through regular assessments and recommendations, as well as by providing field response support and capacity-building with its new permanent European Health Task Force [4]. Surveillance will remain a cornerstone of the Centre’s strategy, as a main source of critical information for public health decision-making. As well as continuing its close cooperation with EU countries, the European Commission and other partners, the agency will also expand its relationships with other international health organisations and major Centres for Disease Control (CDCs). In May 2024, ECDC published a joint framework with four other EU agencies to support the implementation of the ‘One Health’ agenda [5], meaning we will work much more closely across sectors: human, animal and plant health, and food safety, to address the climate crisis, and environmental sustainability.

The agency’s Foresight Programme [6] will allow us to look beyond the immediate horizon to anticipate long-term future developments and potential disruptions, while mathematical modelling will assess the expected short- and medium-term evolution of public health threats and the impact of interventions. All this will guide us in the prioritisation of resources so we can produce relevant work with the greatest impact.

In coming years, ECDC will also prioritise the prevention of antimicrobial resistance and healthcare-associated infections, and support EU countries and the European Commission in reaching the Sustainable Development Goals targets on ending HIV, tuberculosis and hepatitis.

An additional key objective will be to improve the effectiveness of digitalised and integrated infectious disease surveillance, and capacity-building in countries. We need to use all the technological tools available, including artificial intelligence and other innovations, to support rather than hamper our work and to ensure that we are prepared for the next pandemic.

We need collaborative leadership to invigorate and renew confidence in scientific evidence and independent public institutions. Citizens should know, value, and most importantly trust ECDC. In that way, we can make a greater contribution to protecting the population overall, and specifically the most vulnerable members of society – and directly impact people’s lives for the better.

## Note

Pamela Rendi-Wagner is the Director of the European Centre for Disease Prevention and Control (ECDC).
